# High-Intensity Interval Training Ameliorates High-Fat Diet-Induced Metabolic Disorders via the Cyclic GMP-AMP Synthase-Stimulator of Interferon Gene Signaling Pathway

**DOI:** 10.3390/ijms241813840

**Published:** 2023-09-08

**Authors:** Zhiwen Hu, Xi Li, Yangjun Yang, Zhe Zhang, Shuzhe Ding

**Affiliations:** 1 Key Laboratory of Adolescent Health Assessment and Exercise Intervention, Ministry of Education, East China Normal University, Shanghai 200241, China; hzw112514@163.com (Z.H.); lixi989508060506@163.com (X.L.); yjyang98@163.com (Y.Y.); 2 College of Physical Education and Health, East China Normal University, Shanghai 200241, China

**Keywords:** cGAS-STING signaling pathway, high-intensity interval training, moderate-intensity continuous training, metabolic disorders, high-fat diet

## Abstract

Metabolic diseases are growing in prevalence worldwide. Although the pathogenesis of metabolic diseases remains ambiguous, the correlation between cyclic GMP-AMP synthase (cGAS)-stimulator of interferon gene (STING) and metabolic diseases has been identified recently. Exercise is an effective intervention protecting against metabolic diseases, however, the role of the cGAS-STING signaling pathway in this process is unclear, and the effect and mechanism of different exercise intensities on metabolic disorders are still unknown. Thus, we explored the association between exercise to ameliorate HFD-induced metabolic disorders and the cGAS-STING signaling pathway and compared the effects of high-intensity interval training (HIIT) and moderate-intensity continuous training (MICT). Male C57BL/6 mice (6–8 weeks old) were fed HFD for 8 weeks to establish a metabolic disease model and were subjected to 8-week MICT or HIIT training. Glucose tolerance tests (GTT) and insulin tolerance tests (ITT) were used to assess glucose metabolism. Serum triglyceride (TG) and total cholesterol (TC) were measured to evaluate lipid metabolism. Oil red staining was used to observe the lipid droplets in the gastrocnemius muscle. An enzyme-linked immunosorbent assay was used to detect the serum inflammatory factors IL-6 and IFN-β. The protein expression of the cGAS-STING signaling pathway was detected by the Wes^TM^ automatic protein expression analysis system. We reported that HFD induced metabolic disorders with obesity, abnormal glucolipid metabolism, and significant inflammatory responses. Both HIIT and MICT ameliorated the above adverse reactions, but MICT was superior to HIIT in improving glucolipid disorders. Additionally, HIIT significantly increased the expression of STING protein, as well as the phosphorylation of TBKI and the ratio of p-IRF3/IRF3. MICT only increased the expression of STING protein. Our findings suggest that HIIT may alleviate HFD-induced metabolic disorder phenotype through the cGAS-STING signaling pathway. However, the improvement of MICT on metabolic disorder phenotype is less associated with the cGAS-STING pathway, which needs to be further explored.

## 1. Introduction

The incidence of metabolic diseases such as obesity, type 2 diabetes mellitus (T2DM), and cardiovascular diseases is increasing year by year due to population aging and poor lifestyle. Therefore, it is urgent to study the pathological mechanisms and prevention strategies of metabolic diseases. Notably, in recent years, research on metabolic diseases has increasingly focused on the field of immune inflammation. Obesity caused by excessive energy intake is associated with a low-grade inflammatory response in multiple tissues of the body, increasing the risk of metabolic diseases such as T2DM and non-alcoholic fatty liver disease (NAFLD) [1].

Numerous studies have shown that the cGAS-STING signaling pathway, one of the pattern recognition receptors (PRRs) of innate immunity, plays a pivotal role in the prevention and treatment of metabolic diseases, and this pathway may be a new target to combat metabolic diseases such as obesity and T2DM [2]. Cyclic GMP-AMP synthase (cGAS) activates the stimulator of the interferon gene (STING) by sensing external pathogens or its own double-stranded DNA (dsDNA) [3,4]. The activated STING is transported between the endoplasmic reticulum and Golgi [5] and at the same time recruits and activates intracellular TANK binding kinase 1 (TBK1) [6,7]. In turn, TBK1 phosphorylates interferon regulatory factor3 (IRF3) [8], which activates it to form a dimer and translocate to the nucleus, thereby regulating the production of downstream IFN-β and various other inflammatory cytokines (e.g., IL-6, TNF-α, etc.) [9]. Therefore, the dysregulation of the cGAS-STING signaling pathway may lead to excessive inflammatory response and then participate in the development of various metabolic diseases such as T2DM, tumors, and neurodegenerative diseases. Studies have shown that activation of the cGAS-STING signaling pathway can inhibit tumor development [10]. On the other hand, continued activation leads to a chronic inflammatory environment that promotes further tumor growth and metastasis [11]. Therefore, to maintain immune homeostasis, the cGAS-STING signaling pathway must be precisely regulated in the organism.

On the other hand, exercise is an effective intervention to combat metabolic diseases. Lack of exercise contributes to obesity, cardiovascular disease, T2DM, and cancer. Regular physical activity has long been shown to undoubtedly have beneficial or preventive effects on these diseases, and additionally delay depressive symptoms, neurodegeneration (including Alzheimer’s disease), and general aging [12,13,14,15]. Numerous studies have shown that a series of metabolic diseases are accompanied by increased levels of inflammation, and the chronic low-grade inflammatory response caused by such metabolic disorders, also known as metabolic inflammation, is a key pathogenic factor in metabolic diseases [16]. Studies have shown that exercise can improve disease-related metabolic inflammation through immune signaling pathways, which may be an important way to promote health and prevent and treat diseases [17,18,19,20,21]. Some studies have shown that exercise activation of the cGAS-STING signaling pathway can reduce the risk of lung cancer [22]. Therefore, the cGAS-STING signaling pathway may be related to exercise against metabolic diseases. However, the specific mechanism is unclear, and whether the amelioration of metabolic disorders through exercise is related to the regulation of the cGAS-STING signaling pathway remains to be further validated. In addition, it is unclear whether different exercise intensities produce different effects.

At present, the common exercise interventions for metabolic diseases are mainly moderate-intensity continuous training (MICT) and high-intensity interval training (HIIT). HIIT usually involves repeated bouts of high-intensity exercise interspersed with short periods of low-intensity exercise or rest, while MICT is performed in a continuous manner and at lower intensities than HIIT [23]. Although traditional MICT effectively enhances metabolic function, it is characterized by a single exercise mode and a time-consuming nature. In recent years, HIIT has gained popularity as a short-time and high-efficiency exercise method. Numerous studies have demonstrated the significant benefits of HIIT [24], leading to its gradual application in intervention research on metabolic diseases [25]. Therefore, it is necessary to compare the effects of different exercise intensities on metabolic diseases.

Therefore, we established a metabolic disease model by feeding C57BL/6 mice on a high-fat diet (HFD), which were subjected to 8-week MICT or HIIT so as to explore the effects and mechanism of different exercise intensity on HFD-induced metabolic disorders.

## 2. Results

### 2.1. HFD Causes Obesity and Abnormal Glucolipid Metabolism in Mice

**Figure 1:**
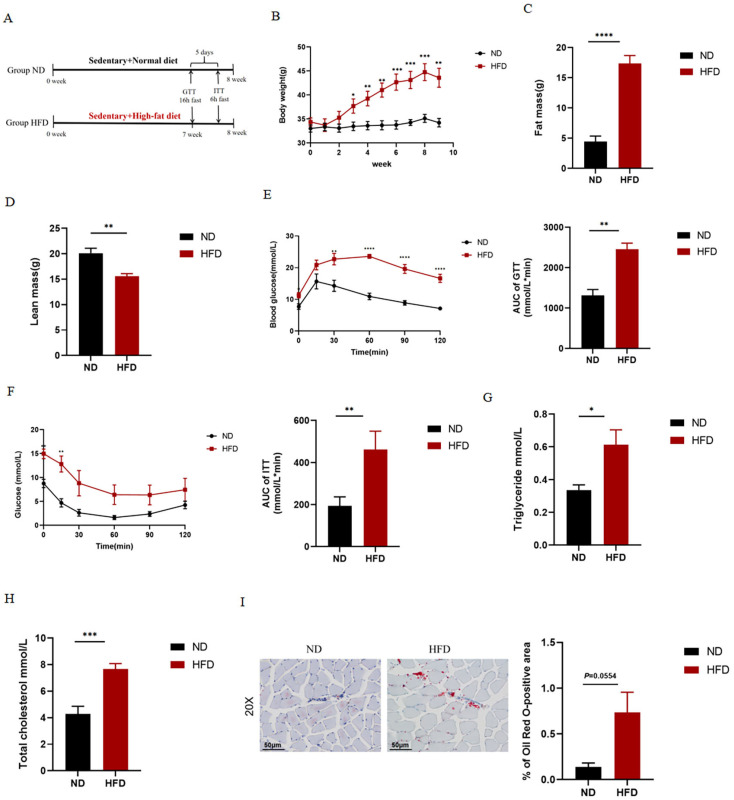
Effects of HFD on metabolic indicators. (**A**) Experimental timeline. (**B**) Body weight. (**C**,**D**) Body composition. (**E**) Glucose tolerance test (GTT). (**F**) Insulin tolerance test (ITT). (**G**,**H**) Serum triglyceride (TG) and total cholesterol (TC) levels. (**I**) Representative images (scale bar: 50 μm; Magnification: 20×) and quantitative analysis of Oil Red O (ORO) staining of gastrocnemius muscle. Data are presented as mean ± SEM. (*n* = 7 for ND, *n* = 7 for HFD). * *p* < 0.05, ** *p* < 0.01, *** *p* < 0.001, **** *p* < 0.0001 vs. Group ND.

As shown in Figure 1, we examined relevant metabolic indicators in normal diet and HFD mice. In the present study, as expected, HFD significantly increased body weight (Figure 1B) and fat mass (Figure 1C), disturbed glucose tolerance (Figure 1E) and insulin sensitivity (Figure 1F), and significantly increased serum TG (Figure 1G) and TC (Figure 1H) levels. As shown in Figure 1, we also observed that HFD resulted in substantial lipid deposition in the gastrocnemius muscle of mice.

### 2.2. The cGAS-STING Signaling Pathway Is Not the Underlying Cause of Metabolic Disorders in HFD Mice

**Figure 2:**
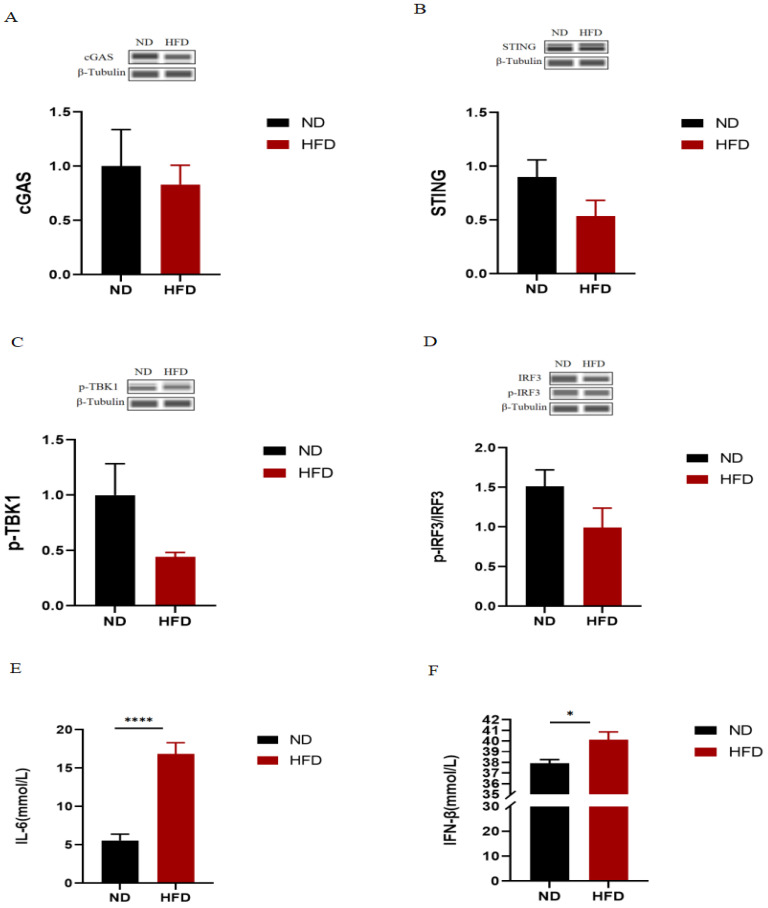
Effects of HFD on the cGAS-STING signaling pathway in skeletal muscle. (**A**–**D**) Expression of the cGAS-STING signaling pathway-related proteins (*n* = 3 for each group). (**E**,**F**) Serum IL-6 and IFN-β levels (*n* = 7 for ND, *n* = 7 for HFD). All data are presented as mean ± SEM. * *p* < 0.05, **** *p* < 0.0001 vs. Group ND.

To further explore whether HFD-induced metabolic disorders are related to the cGAS-STING signaling pathway, We examined the expression of proteins related to the cGAS-STING signaling pathway in the skeletal muscle and the serum levels of inflammatory markers IL-6 and IFN-β in normal diet and HFD mice. Unexpectedly, there were no significant differences in the expression of all proteins between the ND and HFD groups. Additionally, as expected, we found that serum levels of IL-6 and IFN-β were significantly increased in HFD mice (Figure 2E,F).

### 2.3. MICT and HIIT Ameliorate HFD-Induced Obesity and Abnormal Glucolipid Metabolism

**Figure 3:**
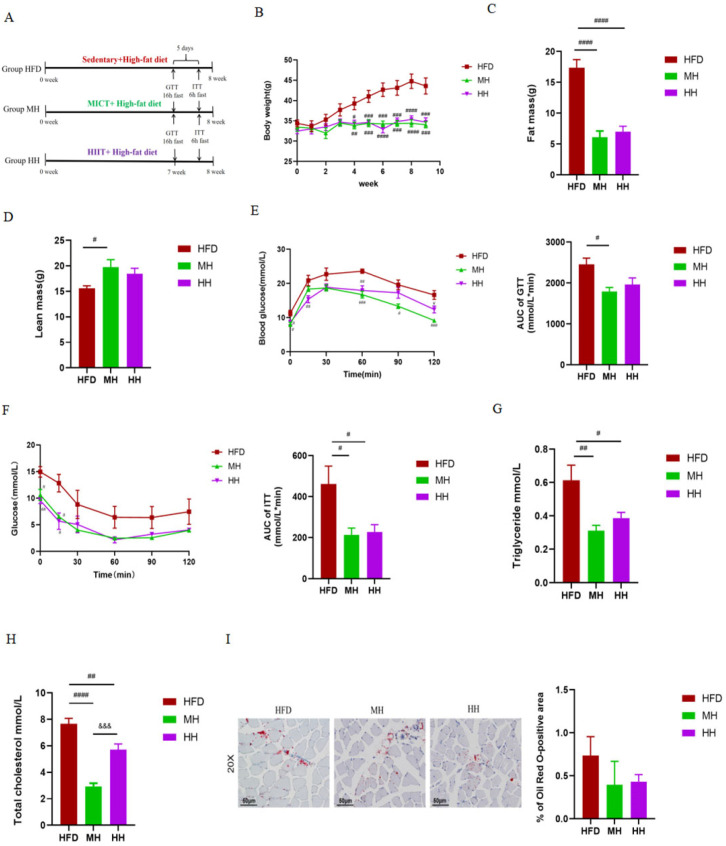
Effects of MICT and HIIT on metabolic disorders in HFD mice. (**A**) Experimental timeline. (**B**) Body weight. (**C**,**D**) Body composition. (**E**) Glucose tolerance test (GTT). (**F**) Insulin tolerance test (ITT). (**G**,**H**) Serum triglyceride (TG) and total cholesterol (TC) levels. (**I**) Representative images (scale bar: 50 μm; Magnification: 20×) and quantitative analysis of Oil Red O (ORO) staining of gastrocnemius muscle. Data are presented as mean ± SEM. (*n* = 7 for HFD, *n* = 7 for MH, *n* = 8 for HH). # *p* < 0.05, ## *p* < 0.01, ### *p* < 0.001, #### *p* < 0.0001 vs. Group HFD, and &&& *p* < 0.001 vs. Group MH.

In the present study, as expected, both MICT and HIIT interventions significantly reversed the HFD-induced metabolic disorders. As shown in Figure 3, MICT and HIIT significantly reduced body weight (Figure 3B) and fat mass (Figure 3C), improved glucose tolerance (Figure 3E) and insulin sensitivity (Figure 3F), and significantly reduced serum TG (Figure 3G) and TC (Figure 3H) levels in HFD mice. Compared with HIIT, MICT was more effective in increasing lean body mass (Figure 3D) and improving glucolipid disorders (Figure 3E,G,H). As shown in Figure 3I, we also observed that MICT and HIIT reduced lipid deposition in the skeletal muscle of HFD mice.

### 2.4. HIIT Ameliorates HFD-Induced Metabolic Disorders via the cGAS-STING Pathway

**Figure 4:**
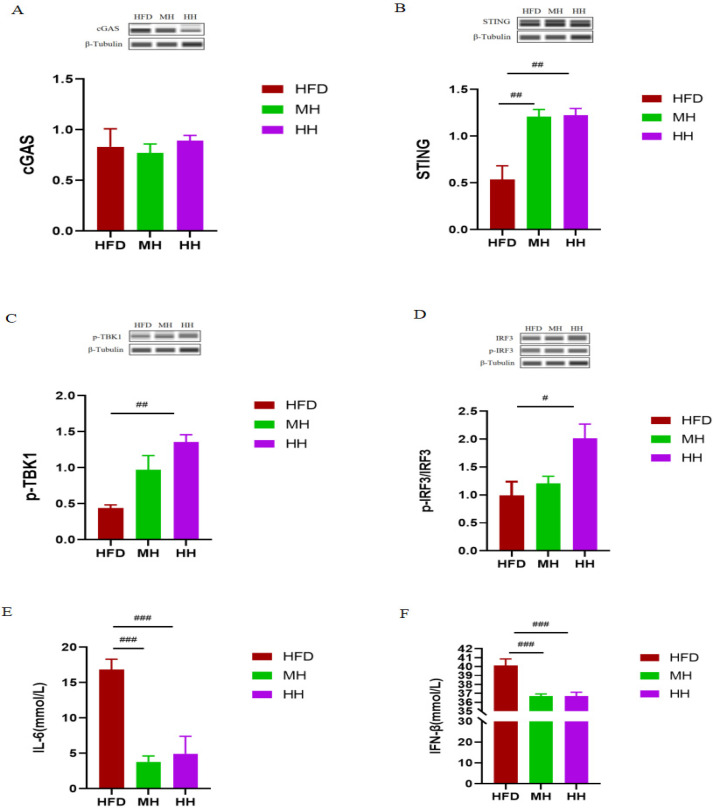
Effects of HIIT and MICT on the cGAS-STING signaling pathway in skeletal muscle of HFD mice. (**A**–**D**) Expression of cGAS-STING signaling pathway-related proteins (*n* = 3 for each group). (**E**,**F**) Serum IL-6 and IFN-β levels (*n* = 7 for HFD, *n* = 7 for MH, *n* = 8 for HH). All data are presented as mean ± SEM. # *p* < 0.05, ## *p* < 0.01, ### *p* < 0.001 vs. Group HFD.

To further verify whether the improvement of HIIT and MICT on HFD-induced metabolic disorders is related to the cGAS-STING signaling pathway, we examined the expression of proteins related to the cGAS-STING signaling pathway in the skeletal muscle and the serum levels of inflammatory markers IL-6 and IFN-β in the mice of the HFD, MH, and HH groups. Our results showed that HIIT significantly increased the expression of STING protein (Figure 4B), as well as the phosphorylation of TBKI (Figure 4C) and the ratio of p-IRF3/IRF3 (Figure 4D). In addition, as expected, HIIT significantly reduced serum IL-6 (Figure 4E) and IFN-β (Figure 4F) levels in HFD mice. As for MICT, our results showed that MICT significantly increased the expression of STING protein (Figure 4B). Unexpectedly, there were no significant differences in the expression of other proteins between the MH and HFD groups. Interestingly, we also found that MICT significantly reduced serum IL-6 (Figure 4E) and IFN-β (Figure 4F) levels in HFD mice.

## 3. Discussion

In recent years, there has been an increasing interest in studying the different effects of HIIT and MICT exercise modalities on metabolic diseases [24,25,26,27,28,29]. High-fat feeding is widely used to make animal models of metabolic diseases, and a large number of research results have been achieved [30]. The prevalence of obesity gradually increases with a high-fat and high-energy diet, leading to metabolic abnormalities such as excessive lipid deposition causing hyperlipidemia and NAFLD. Additionally, it inhibits insulin signaling, resulting in insulin resistance and T2DM. Adipocyte dysfunction can also cause systemic inflammation leading to conditions like high blood pressure and cardiovascular disease. Here, in an experimental model, we demonstrated HFD-induced obesity, glucose intolerance, and reduced insulin sensitivity, as well as abnormal serum lipid and inflammatory factor levels. Thus, as expected, 8 weeks of HFD resulted in metabolic disorders. However, exercise training, particularly HIIT, may reverse these adverse changes by activating the cGAS-STING signaling pathway. These findings were consistent with reduced body fat, improved glucose and insulin sensitivity, reduced serum lipid levels, and inflammatory markers.

There has been substantial research evidence that exercise interventions have a better modulating effect on metabolic disorders [31]. MICT and HIIT are widely used in the health management of metabolic syndrome, diabetes, hypertension, and other metabolic diseases [24,25,26,27,28,29], but the question of which intervention mode produces better results in patients with metabolic diseases remains controversial, and no consensus has been reached [26,27,32,33,34,35]. The present study examined the differential effects of HIIT and MICT on HFD-induced metabolic disorders. We found that HIIT and MICT showed similar effects in reducing body weight, fat content, and serum inflammatory markers in HFD mice. Furthermore, MICT was more effective than HIIT in increasing lean body mass, improving glucose intolerance and insulin sensitivity, as well as ameliorating dyslipidemia in HFD mice. However, in theory, HIIT is more effective in improving glucose regulation [36]. The mechanisms by which exercise regulates glycemic control and improves blood glucose suggest that exercise improves muscle insulin sensitivity and post-exercise glucose allocation by enhancing glucose transport in muscle tissue while improving intra-muscle cell metabolism [37]. Whereas all these processes are related to the intensity of exercise, there is not enough experimental evidence to support a better effect of HIIT on glucose regulation [36,38]. Our present findings showed that a dyslipidemia phenotype occurs following HFD and lack of exercise; moreover, we observed substantial lipid deposition in the skeletal muscle of HFD mice. In MICT exercise mode, skeletal muscle consumes more lipids from circulation due to its lower exercise intensity; thus, MICT has a stronger effect on changing the systemic lipid levels, such as serum TG and TC than HIIT.

In recent years, numerous studies have shown that the cGAS-STING signaling pathway plays a pivotal role in the prevention and treatment of metabolic diseases, and this pathway may be a new target to combat metabolic diseases such as obesity and T2DM [2]. To further investigate whether HFD-induced metabolic disorders are related to the skeletal muscle cGAS-STING signaling pathway, we examined the expression of key proteins of this pathway. However, we had not yet found a correlation between HFD-induced metabolic disorders and the cGAS-STING signaling pathway in skeletal muscle. This suggests that this pathway may not be the fundamental cause of metabolic disorders. Previous studies have shown that the development of metabolic disorders is associated with multiple triggers, such as diet, environment, and exercise, which involve complex molecular mechanisms, including dysregulation of glucose and insulin signaling, mitochondrial dysfunction, and oxidative stress [39,40,41]. Although a significant inflammatory response was also found in HFD mice, it was less associated with the skeletal muscle cGAS-STING signaling pathway, which may be generated by other inflammatory signaling pathways or stimulated by other immune organs. The specific mechanism remains to be further explored. In fact, although there was no statistical difference in the expression of the cGAS-STING pathway in HFD mice, we found that the expression of this pathway was overall lower, and with the further aggravation of metabolic disorders in HFD mice, the expression of the cGAS-STING pathway was not up-regulated. Similarly, studies have shown that cGAS-STING pathway expression is decreased in diabetic cardiomyopathy mice to promote the progression of type 1 diabetes [42]. In addition, the expression of the cGAS-STING pathway is also inhibited in cardiomyocytes in a high-glucose environment, and its activation by agonists can protect cardiomyocytes by suppressing inflammation [42]. Therefore, we suggest that the cGAS-STING pathway may have a protective effect. However, some studies have also shown that upregulation of the cGAS-STING pathway leads to aggravation of atherosclerosis and NAFLD [43,44,45]. Overall, the cGAS-STING pathway exerts a complex influence on the development of different metabolic diseases, not only through the promotion of inflammatory damage but also with protective functions in some respects. Given the different effects of signaling leading to disease under various conditions, deterioration or amelioration is possible. The current literature has demonstrated that the cGAS-STING pathway plays an important role in different metabolic diseases related to inflammation. However, the present study found that HFD-induced metabolic abnormalities were less associated with the skeletal muscle cGAS-STING signaling pathway, so further studies on the cGAS-STING pathway and metabolic diseases need to be explored.

Although we found that the skeletal muscle cGAS-STING pathway was not the underlying cause of metabolic disorders, whether it plays a role in the amelioration of metabolic disorders is unknown, and we performed further validation. Our results showed that HIIT significantly increased the expression of STING protein, as well as the phosphorylation of TBKI and the ratio of p-IRF3/IRF3. In addition, our study found that 8-week HIIT significantly ameliorated HFD-induced upregulation of serum IL-6 and IFN-β. This suggests that long-term HIIT may exert immune regulation and play an anti-inflammatory role by activating the cGAS-STING signaling pathway, thus effectively alleviating metabolic disorder phenotype. These results further verified the significance of the cGAS-STING signaling pathway in regulating the inflammatory response in metabolic diseases, but there is no evidence to confirm whether the cGAS-STING signaling pathway is necessary for exercise to ameliorate metabolic inflammation accompanied by metabolic diseases. Regrettably, MICT only increased the expression of STING protein, with no significant differences in the expression of other proteins in HFD mice. This suggests that the improvement of metabolic disorder phenotype by MICT is less associated with the skeletal muscle cGAS-STING signaling pathway. However, we found that MICT also significantly reduced the expression levels of serum IL-6 and IFN-β in HFD mice, which may be due to the activation of other STING-induced immune signaling pathways by MICT, and the specific mechanism of its role needs to be further explored. STING is a key adaptor protein of the innate immune system and has the characteristics of inflammatory molecules [5]. Up-regulation of STING expression causes the activation of related signaling pathways, such as the classical NF-κB signaling pathway, which is closely related to the development of various inflammatory diseases. Studies have confirmed that STING can act in metabolic regulation independent of cGAS [46,47,48]. Gezhe [22] found that aerobic endurance exercise activated the cGAS-STING signaling pathway while HIIT did not in lung cancer tissue, which was contrary to our findings. This may be caused by different mice models and detected tissues. However, in general, they all show that the activation of the cGAS-STING signaling pathway is closely related to exercise intensity. Therefore, how to improve innate immune function through reasonable exercise is the research direction to enhance the function of the body’s sports system and improve many chronic inflammation-related diseases.

The specific mechanism of HIIT activation of cGAS-STING signaling pathway in skeletal muscle of HFD mice is speculated to be related to exercise-induced muscle damage. Studies have shown that exercise-induced muscle damage may affect the function of the immune system for several days after exercise ends [49]. It may be due to the damage of skeletal muscle induced by HIIT, which leads to the destruction of mitochondrial structure and function [50], and then some mitochondrial DNA (mtDNA) is released into the cytoplasm or extracellular interstitium. As a key molecule to activate damage-associated molecular patterns, mtDNA is recognized by the pattern recognition receptor cGAS, which further activates the cGAS-STING signaling pathway, and finally regulates the expression of TNF-α, IL-6, IL-1β and other pro-inflammatory factors after skeletal muscle injury. It has also been suggested that activated mitophagy in muscle cells can reduce local inflammatory response through the cGAS-STING signaling pathway [51]. Tuan et al. [51] found that myocardial mitochondrial damage in mice after exhaustive exercise triggered mitophagy to remove damaged mitochondria, reduce the release of mtDNA, and thus slow down the inflammatory signal and interferon signal activated by STING. Therefore, the cGAS-STING signaling pathway may be involved in the inflammatory response process of exercise-induced muscle damage.

In fact, the cGAS-STING signaling pathway, initially identified for its role in the innate immune response against viral infections and DNA damage, has recently been implicated in the regulation of inflammation and metabolism, as confirmed by our study. This raises the intriguing possibility that the cGAS-STING signaling pathway might also contribute to the development of metabolic disorders induced by factors other than high-fat diets, such as genetic factors or environmental triggers. It is well known that metabolic disorders have become a global health problem. In addition to high-fat diets, metabolic dysregulation can also arise due to other factors such as genetics and environment [52]. Environmental triggers like oxidative stress and DNA damage, which are known contributors to metabolic disorders, have also been reported to activate the cGAS-STING pathway [53], suggesting its potential involvement in the pathogenesis of metabolic dysregulation beyond HFD-induced conditions. Importantly, several common features often accompany metabolic disorders caused by different factors, including glucose intolerance, insulin resistance, dyslipidemia, and inflammatory responses, which were consistent with our findings. Among them, metabolic inflammation is the hallmark of metabolic diseases [16,54], accompanied by infiltration and activation of immune cells along with increased inflammatory cytokines that impair insulin signaling and disrupt systemic metabolic homeostasis [16,55,56]. Inflammatory signaling pathways (such as JNK and IKK) and inflammatory cytokines (such as TNF-a and IL-1b) have been reported to be involved in the pathogenesis of metabolic diseases [16,57]. Therefore, exploring the impact of inflammation-related signaling pathways and inflammatory cytokines is an essential step towards understanding the pathogenesis of metabolic disorders. The present study was based on a metabolic disorder model and investigated the cGAS-STING signaling pathway in light of these considerations. Although extensive research has been conducted on the role of the cGAS-STING signaling pathway in metabolic diseases, its precise association with metabolic disorders other than those induced by HFD remains to be clarified. However, it is becoming increasingly evident that this pathway may extend beyond HFD-induced diseases. Both genetic and environmental triggers appear to be potential factors that can influence the cGAS-STING pathway, leading to chronic inflammation and metabolic dysfunction. Therefore, further studies are needed to fully elucidate the complex molecular mechanisms linking the cGAS-STING pathway and inflammation in diverse etiological contexts of metabolic disorders.

In conclusion, our study confirmed that high-fat dietary feeding successfully constructed a metabolic disorder model. Based on this model, we explored the improvement effect of different exercise intensities on the metabolic abnormal phenotype and its relationship with the skeletal muscle cGAS-STING signaling pathway. Finally, our results revealed that both HIIT and MICT can effectively alleviate HFD-induced metabolic disorders. Interestingly, we found that the skeletal muscle cGAS-STING pathway was not the underlying cause of metabolic disorders, but it did act in the improvement of metabolic disorders. Among them, HIIT could reduce the inflammatory response and reverse the HFD-induced metabolic disorder phenotype by regulating the cGAS-STING signaling pathway in skeletal muscle. However, the improvement of MICT on the metabolic disorder phenotype is less associated with the cGAS-STING pathway, which needs to be further explored.

## 4. Materials and Methods

### 4.1. Animals

Twenty-nine healthy male C57BL/6 mice (6–8 weeks old) were purchased from Shanghai SLAC Laboratory Animal Co., Ltd., Shanghai, China, and housed in a specific-pathogen-free (SPF) environment (humidity 50 ± 5%, temperature 25 ± 2 °C, 12/12 h light/dark cycles). All mice were provided food and water ad libitum. Following 2 weeks acclimation, the mice were randomly divided into four groups: (1) normal diet (ND; *n* = 7); (2) high-fat diet (HFD; *n* = 7); (3) moderate-intensity continuous training (MICT) + HFD (MH; *n* = 7); and (4) high-intensity interval training (HIIT) + HFD (HH; *n* = 8). Group ND was given a normal diet and the other groups were fed with high-fat diet (D12451, Research Diets). The HFD feed consisted of 20.0 kcal% protein, 45.0 kcal% fat, and 35.0 kcal% carbohydrate (energy density 4.7 kcal/g). In this study, all animal experiment procedures were approved by the Animal Ethical Committees of the East China Normal University, Shanghai, China.

### 4.2. Exercise Protocol

We adopted two different intensity treadmill exercise programs: 8-week MICT and HIIT. The specific training protocol was as follows: Animals in the MH and HH groups received a 2-week adaptive training for treadmill running, at 10 m/min for 20 min, 1 time/day, 5 times/week. After the completion of adaptability training, we detected the initial exercise capacity of mice in group HH to determine the appropriate HIIT protocol. The test method was to start the treadmill exercise at a speed of 8 m/min and increase the running speed by 1 m/min every 2 min until exhaustion (mice no longer ran when shocked for more than 10 s). The time and speed (the maximum speed of the initial exercise capacity test) were recorded at this point. Next, the mice of groups MH and HH started the 8-week formal running training. Mice of group MH performed on a treadmill at 14 m/min for 60 min, 1 time/day, 5 times/week, for 8 weeks. Mice of group HH, the first week, performed on a treadmill at 22 m/min (85% of the maximum speed of the exercise capacity test) for 2 min followed by 8.8 m/min (40% of the initial speed of the week) for 2 min, which alternated through 12 cycles, 1 time/day, 5 times/week, and the following weekly initial running speed was increased by 1 m/min from the previous week for a total of 8 weeks [58]. The mice of groups ND and HFD remained sedentary in their cages for the entire duration of the 8-week training program. The exercise protocol is shown in Table 1.

### 4.3. Blood and Tissue Samples Collection

All mice were fasted 12 h and anaesthetized with 4–5% isoflurane. Blood samples were collected from the orbit. Then, mice were sacrificed by cervical dislocation. Gastrocnemius muscle tissue samples were rapidly collected, snap-frozen in liquid nitrogen, and stored at −80 °C for subsequent biochemical analysis. The blood samples were stored in a 4 °C refrigerator overnight and then centrifuged at 1100× *g* for 15 min (4 °C) the next day. The upper serums were taken and packed in Eppendorf tubes (EP tubes) and stored in a −80 °C refrigerator for subsequent analyses.

### 4.4. Glucose Tolerance Test and Insulin Tolerance Test

GTTs were performed 1 week before the end of the exercise intervention. Briefly, GTTs were carried out after a 16 h fast. Mice were injected intraperitoneally with glucose (1 g/kg b.w. i.p., Sigma-Aldrich, St. Louis, MO, USA). Blood samples were obtained from the tail tip at 0, 15, 30, 60, 90, and 120 min for the measurement of glucose levels via a blood glucometer (Accu-Chek Active, Roche Diagnostics GmbH, Mannheim, Germany).

ITT was performed 5 days after GTT. After a 6 h fast, mice received an intraperitoneal injection of insulin (0.75 U/kg b.w. i.p., Novolin R, Novo Nordisk, Copenhagen, Denmark), and blood glucose levels were measured at 0, 15, 30, 60, 90, and 120 min. The area under the curve (AUC) was determined using GraphPad Prism 9.3.0 software (GraphPad Software Inc., San Diego, CA, USA).

### 4.5. Body Composition Analysis

The amounts of body fat and lean body mass were determined in whole live mice. The assessment of total fat and lean mass for each individual animal was acquired by nuclear magnetic resonance (NMR) using the AccuFat MRI system (AccuFat-1050, MAG-MED, Nanjing, China). Briefly, mice were placed in a small animal specimen holder (long cylinder) and kept immobile by the insertion of a tight-fitting plunger into the cylinder. The holder was then lowered into the sample chamber of the machine for measurements. The resulting fat and lean mass measurement was determined in grams, i.e., fat mass (g) and lean mass (g). Fat mass represents the total weight of fat in the body. Lean mass represents the weight of the body’s non-fat components, which are mainly composed of bones, muscles, etc.

### 4.6. Serum Total Cholesterol (TC) and Triglyceride (TG) Test

Serum TG (A110-1-1) and TC (A111-1-1) levels were measured with a commercial triglyceride assay kit and total cholesterol assay kit (Nanjing Jiancheng Bioengineering Research Institute, Nanjing, China), and the whole process followed the manufacturer’s guidelines.

### 4.7. ELISAs of IL-6 and IFN-β

The inflammatory cytokines of IL-6 (EK206) and IFN-β (EK2236) in the serum of mice were determined by the commercial ELISA kits (mice IL-6 kit and mice IFN-β kit, Multi Sciences Biotech, Shanghai, China).

### 4.8. Oil Red O Staining

Oil Red O staining was performed by Wuhan Servicebio Technology Co., Ltd., Wuhan, China. Briefly, the gastrocnemius muscles were extracted and fixed in 4% paraformaldehyde. Gastrocnemius muscle tissues were embedded in an OTC compound, cut into 8 µm-thick sections, and placed on slides. Tissue sections were immersed in 60% isopropanol and incubated in Oil Red O staining solution for 10 min, then differentiated with 60% isopropanol, washed with distilled water twice, counterstained with hematoxylin, and mounted with glycerine gelatin (Servicebio, Wuhan, China). The stained tissue sections were visualized and images were captured using an optical microscope (Olympus Optical Co., Ltd., Tokyo, Japan) and processed using CaseViewer 2.4 (3DHISTECH Ltd., Budapest, Hungary). The lipid deposition in tissue was quantified by using Image Pro Plus 6.0 software (Media Cybernetics, Silver Spring, MD, USA).

### 4.9. Wes Automated Western Blotting System

The protein expression level was detected by Wes^TM^ automatic protein expression analysis system (ProteinSimple, San Jose, CA, USA). Briefly, gastrocnemius muscle tissue (40 mg) was homogenized in RIPA buffer (Beyotime, Shanghai, China) supplemented with protease inhibitor (Beyotime, Shanghai, China), then centrifuged at 12,000× *g* for 20 min at 4 °C. The supernatant was collected and protein concentration was determined using a BCA kit (Beyotime, Shanghai, China). The samples were detected using the Wes^TM^ automatic protein expression analysis system (ProteinSimple, San Jose, CA, USA), and the specific steps were carried out following the instrument manual. The following primary antibodies were used: IFN-β (DF6471, affinity), Phospho-IRF3 (Ser396) (AF2436, affinity), Phospho-TBK1 (Ser172) (5483, CST), IRF3 (DF6895, affinity), TBK1 (ab40676, abcam), cGAS (31659, CST), STING (13647, CST), β-Tubulin (AF7011, Affinity). The internal control for this experiment was β-Tubulin. Image results were analyzed using Compass for SW 6.0.0 software (ProteinSimple, San Jose, CA, USA).

### 4.10. Statistical Analysis

All data in our study are presented as the mean ± SEM and were analyzed by GraphPad Prism 9 software. The comparisons between the two groups were performed by the Student’s *t*-test (unpaired and two-tailed). Multiple comparisons among different groups were analyzed by one-way ANOVA followed by a post hoc Tukey’s test. Statistical significance was defined as *p* < 0.05.

## Figures and Tables

**Table 1:** Exercise training protocol.

	MICT		HIIT				
Weeks	Exercise Speed (m/min)	Exercise Time (min)	Exercise Speed (m/min)	Exercise Time (min)	Rest Speed (m/min)	Rest Time (min)	Number of Repetitions
1	14	60	22	2	8.8	2	12
2	14	60	23	2	9.2	2	12
3	14	60	24	2	9.6	2	12
4	14	60	25	2	10	2	12
5	14	60	26	2	10.4	2	12
6	14	60	27	2	10.8	2	12
7	14	60	28	2	11.2	2	12
8	14	60	29	2	11.6	2	12

## Data Availability

The data that support the findings of this study are available from the corresponding author upon reasonable request.
